# Supplementary material for: Serine hydroxymethyltransferase as a potential target of antibacterial agents acting synergistically with one-carbon metabolism-related inhibitors
Source: Commun Biol. 2022 Jun 23;5:619. doi: 10.1038/s42003-022-03555-x (PMC9223267; doi:10.1038/s42003-022-03555-x)
Supplement: Supplementary file 2 — Supplementary information [file 42003_2022_3555_MOESM2_ESM.pdf]

Supplementary Information

Serine hydroxymethyltransferase as a potential target of antibacterial agents acting synergistically with one-carbon metabolism-related inhibitors

Yuko Makino[1], Chihiro Oe[1], Kazuya Iwama[1], Satoshi Suzuki[1], Akie Nishiyama[1], Kazuya Hasegawa[2], Haruka Okuda[2], Kazushige Hirata[1,3], Mariko Ueno[1], Kumi Kawaji[2], Mina Sasano[2], Emiko Usui[2], Toshiaki Hosaka[4], Yukako Yabuki[4], Mikako Shirouzu[4], Makoto Katsumi[3], Kazutaka Murayama[4,5], Hironori Hayashi[2,6,*] & Eiichi N Kodama[1,2,6,7]

[1]Department of Infectious Diseases, Tohoku University Graduate School of Medicine, 2-1, Seiryo-machi, Aoba-ku, Sendai, Miyagi 980-8575, Japan
[2]Protein Crystal Analysis Division, Japan Synchrotron Radiation Research Institute, 1-1, Sayo-chou, Hyogo, Japan
3Division of Infectious Diseases, International Research Institute of Disaster Science, Tohoku University, 2-1, Seiryo-machi, Aoba-ku, Sendai, Miyagi 980-8575, Japan
4Department of Clinical Laboratory Medicine, Tohoku University Hospital, 1-1, Seiryo-machi, Aoba-ku, Sendai, Miyagi 980-8574, Japan
5Laboratory for Protein Functional and Structural Biology, RIKEN Center for Biosystems Dynamics Research, Yokohama, Japan
6Division of Biomedical Measurements and Diagnostics, Graduate School of Biomedical Engineering, Tohoku University, Sendai, Japan
7Department of Intelligent Network for Infection Control, Tohoku University Graduate School of Medicine, 2-1, Seiryo-machi, Aoba-ku, Sendai, Miyagi 980-8575, Japan
8Tohoku Medical Megabank Organization, Tohoku University, 2-1, Seiryo-machi, Aoba-ku, Sendai, Miyagi 980-8575, Japan
*Correspondence: hhayashi@med.tohoku.ac.jp

Supplementary Information

Table of contents	Page
Supplemental Tables	
Supplementary Table S1	S2
Supplementary Table S2	S3
Supplementary Table S3	S4
Supplementary Table S4	S5
Supplementary Table S5	S6
Supplementary Table S6	S7
Supplemental Figures	
Supplementary Fig. S1	S8
Supplementary Fig. S2	S9
Supplementary Fig. S3	S10
Supplementary Fig. S4	S11
Supplementary Fig. S5	S12
Supplementary Fig. S6	S13
Supplemental Reference	S14

Supplementary Table S1. Primer sequences for qPCR

Primer names	Sequences	Annealing temp. (^{o}C)	Size of PCR product (bp)
ddl-F†	5'- GAGACATTGAATATGCCTTATG -3'	54	550
ddl-R†	5'- AAAAAGAAATCGCACCG -3'		
SHMT-qF	5'- TGCTTATCTCGCTTTGGTAGAAC -3'	60	206
SHMT-qR	5'- CTACGATCAATTTTGGTTGATG -3'		
Fhs-qF	5'- CAACAGTGTGAGTTCATTGTCTG -3'	61	310
Fhs-qR	5'- GAGATTAGCAGATTACGTTGTCAC -3'		
PurN-qF	5'- CTTGGTCGATATAATGAATTGTG -3'	61	184
PurN-qR	5'- CGATTTGATCGTATTAGCTGG -3'		
PurH-qF	5'- AGTACGATCGCATTAGATTTCAC -3'	61	218
PurH-qR	5'- CGATTACTGACAGTAGACTTTGTG -3'		
FolD-qF	5'- CTACATCTATCACTACAGCACCTG -3'	61	247
FolD-qR	5'- ATGTTTGAAGCTTATTCTATCGATC -3'		
MetE-qF	5'- CGACAAGAGATTCTATCTAATGATGG -3'	61	158
MetE-qR	5'- CAATTTGTATCATCGAATTGCAG -3'		
GcvH-qF	5'- GAACCTCTGATCGAAGTTGAAG -3'	61	163
GcvH-qR	5'- GATCAACATCTTTGAAACTTAAGATC -3'		
ThyA-qF	5'- GACCACAATATTTAGAACAGAAGTATG -3'	58	149
ThyA-qR	5'- TCTTCTTTTGCCATACAATGG -3'		
DHFR-qF	5'- GAACATACAATGGATCGAATTTTAG -3'	61	151
DHFR-qR	5'- CATCAATCGAATGCATTACTTC -3'		

†The primer sequences were reported previously.

Supplementary Table S2. Cell cytotoxicity of folate-mediated 1C metabolism inhibitors.

Compounds	Caski		Hep-2		Calu-3	
	CC_{50} (μM) (pCC_{50} ± S.E.)	S.I.†	CC_{50} (μM) (pCC_{50} ± S.E.)	S.I.†	CC_{50} (μM) (pCC_{50} ± S.E.)	S.I.†
(+)-SHIN-1	> 2 (< 5.7)	> 45454	> 2 (< 5.7)	> 45454	> 2 (< 5.7)	> 45454
SHMT-IN-2	28 (4.5 ± 0.0)	10	31 (4.5 ± 0.0)	11	46 (4.3 ± 0.0)	16
PMX	0.20 (6.7 ± 0.1)	2800	5.6 (5.3 ± 0.2)	14000	> 100 (< 4.0)	> 2000000
MTX	0.018 (7.8 ± 0.1)	5	0.13 (6.9 ± 0.2)	62	> 100 (< 4.0)	> 27027
SER	20 (4.7 ± 0.0)	1	24 (4.6 ± 0.0)	1	3.2 (5.5 ± 0.0)	0.2
TMP	≥ 100‡ (≤ 4.0)	≥ 34482	> 100 (< 4.0)	≥ 34482	> 100 (< 4.0)	≥ 34482

CC_{50}, which is the concentration of a test compound that decreases cell viability by 50%, is the range determined from three independent experiments. pCC_{50} is log-transformed CC_{50}. All data represent the mean ± standard deviation, n = 3–4. †Selectivity index (SI) is the ratio of each compound's CC_{50} against each cell line and the EC_{50} against *E. faecium* (as described in Table 1). ‡Two of four CC_{50} values were over 100 μM and the others were ~100 μM. Caski: a cervical cancer cell line infected by human papillomavirus type 16; Hep-2: a human laryngeal cancer cell line; Calu-3: a human lung cancer cell line. PMX: pemetrexed; MTX: methotrexate; SER: sertraline;

Supplementary Table S3. Amino acid sequences of the 115- and 343-loops in SHMT from different species.

	a.a. sequence	
	115-loop	343-loop
*efm*SHMT:	MDLSAGGHLTHGS-----PVNFSGKTYH	NSIPFETLSPFKTSG
*ec*SHMT:	MNLAHGGHLTHGS-----PVNFSGKLYN	NSVPNDPKSPFVTSG
*pv*SHMT:	MHLCSGGHLTHGFFDEKKKVSITSDMFE	NTIPSDVD-CVSPSG
*hu*SHMT2:	LDLPDGGHLTHGYMSDVKRISATSIFFE	NTCPGDRS--AITPG

Amino acid sequences of the 115-loop and 343-loop. The 115-loop was constructed from M115 to H137 (*ec*SHMT, M119–N141; *pv*SHMT, M122–E149; *hu*SHMT, L164–E191). Loop-343 includes amino acids N343–G357 (*ec*SHMT, N347–G361; *pv*SHMT, N356–G369; *hu*SHMT, N410–G423). Red letters indicate conserved amino acids among the four SHMTs.

Supplementary Table S4. EC_{50} and C.I. values for the (+)-SHIN-1 and nucleoside-analogue

(+)-SHIN-1	5-FdU			5-FdU	(+)-SHIN-1		
Conc. (nM) (pConc.)	EC_{50} (nM) (pEC_{50} ± S.E.)	F.I.[*]	C.I.[†]	Conc. (nM) (pConc.)	EC_{50} (nM) (pEC_{50} ± S.E.)	F.I.[*]	C.I.[†]
0 (-)	20 (7.7 ± 0.0)	1.0	1.0	0 (-)	0.045 (10.3 ± 0.2)	1.0	1.0
0.00002 (13.7)	3.0 (8.5 ± 0.0)	0.2	0.15	0.01 (11.0)	0.0058 (11.0 ± 0.2)	0.1	0.13
0.0002 (12.7)	1.8 (8.7 ± 0.1)	0.1	0.092	0.1 (10.0)	0.0068 (11.2 ± 0.2)	0.1	0.16
0.002 (11.7)	0.63 (9.2 ± 0.1)	0.03	0.031	1 (9.0)	0.0093 (11.2 ± 0.1)	0.2	0.27
(+)-SHIN-1	5-FUrd			5-FUrd	(+)-SHIN-1		
Conc. (nM) (pConc.)	EC_{50} (nM) (pEC_{50} ± S.E.)	F.I.[*]	C.I.[†]	Conc. (nM) (pConc.)	EC_{50} (nM) (pEC_{50} ± S.E.)	F.I.[*]	C.I.[†]
0 (-)	72 (7.1 ± 0.1)	1.0	1.0	0 (-)	0.036 (10.4 ± 0.4)	1.0	1.0
0.00002 (13.7)	11 (8.0 ± 0.2)	0.2	0.15	0.01 (11.0)	0.025 (10.6 ± 0.3)	0.7	0.69
0.0002 (12.7)	3.8 (8.4 ± 0.3)	0.05	0.057	0.1 (10.0)	0.014 (10.8 ± 0.3)	0.4	0.41
0.002 (11.7)	3.9 (8.4 ± 0.3)	0.05	0.11	1 (9.0)	0.013 (10.9 ± 0.3)	0.3	0.37

The 50% effective concentration (EC_{50}) is the range determined from at least three independent experiments. pEC_{50} , and pConc are log-transformed EC_{50}, and Conc., respectively. All data represent the mean ± standard deviation, n = 4–6. [†]Combination index (C.I.) is calculated from the following equation: C.I. = $D_A/D_{50,A}+D_B/D_{50,B}$. Where D_A and D_B are the concentrations of compound A and B that achieve a 50% effective concentration under drug combination, and $D_{50,A}$ and $D_{50,B}$ denote the EC_{50} values under monotherapy. C.I. < 1.0 denotes synergism, C.I. = 1.0 denotes an additive effect and C.I. > 1.0 denotes antagonism.

Supplementary Table S5. EC_{50} and C.I. values for the MTX and nucleoside analogue combinations.

MTX	5-FdU			5-FdU	MTX		
Conc. (nM) (pConc.)	EC_{50} (nM) (pEC_{50} ± S.E.)	F.I.*	C.I.†	Conc. (nM) (pConc.)	EC_{50} (nM) (pEC_{50} ± S.E.)	F.I.*	C.I.†
0 (-)	21 (7.7 ± 0.0)	1.0	1.0	0 (-)	2.2 (8.7 ± 0.0)	1.0	1.0
0.001 (12.0)	4.8 (8.3 ± 0.1)	0.2	0.23	0.01 (11.0)	1.1 (8.9 ± 0.1)	0.5	0.53
0.01 (11.0)	4.6 (8.3 ± 0.1)	0.2	0.22	0.1 (10.0)	1.1 (9.0 ± 0.1)	0.5	0.49
0.1 (10.0)	3.8 (8.4 ± 0.1)	0.2	0.22	1 (9.0)	0.54 (9.3 ± 0.2)	0.2	0.24

MTX	5-FUrd			5-FUrd	MTX		
Conc. (nM) (pConc.)	EC_{50} (nM) (pEC_{50} ± S.E.)	F.I.*	C.I.†	Conc. (nM) (pConc.)	EC_{50} (nM) (pEC_{50} ± S.E.)	F.I.*	C.I.†
0 (-)	84 (7.1 ± 0.1)	1.0	1.0	0 (-)	1.6 (8.8 ± 0.1)	1.0	1.0
0.001 (12.0)	31 (7.5 ± 0.1)	0.4	0.42	0.01 (11.0)	0.66 (9.1 ± 0.3)	0.7	0.37
0.01 (11.0)	22 (7.7 ± 0.2)	0.3	0.26	0.1 (10.0)	0.40 (9.4 ± 0.3)	0.3	0.27
0.1 (10.0)	4.3 (8.3 ± 0.5)	0.07	0.077	1 (9.0)	0.10 (9.9 ± 0.8)	0.05	0.11

The 50% effective concentration (EC_{50}) is the range determined from at least three independent experiments. pEC_{50} , and pConc are log-transformed EC_{50}, and Conc., respectively. All data represent the mean ± standard deviation, n = 4–6. †Combination index (C.I.) was calculated as C.I. = $D_A/D_{50,A} + D_B/D_{50,B}$, where D_A and D_B are the concentrations of compounds A and B, respectively, that achieve a 50% effective concentration under drug combinations, and $D_{50,A}$ and $D_{50,B}$ denote the EC_{50} values under monotherapy. C.I. < 1.0 denotes synergism, C.I. = 1.0 denotes an additive effect and C.I. > 1.0 denotes antagonism.

Supplementary Table S6. EC_{50} and C.I. values for MTX and (+)-SHIN-1

(+)-SHIN-1	MTX			MTX	(+)-SHIN-1		
Conc. (nM) (pConc.)	EC_{50} (nM) (pEC_{50} ± S.E.)	F.I.*	C.I.†	Conc. (nM) (pConc.)	EC_{50} (nM) (pEC_{50} ± S.E.)	F.I.*	C.I.†
0 (-)	1.6 (8.8 ± 0.1)	1.0	1.0	0 (-)	0.031 (10.5 ± 0.3)	1.0	1.0
0.00002 (13.7)	0.51 (9.2 ± 0.2)	0.3	0.33	0.01 (11.0)	0.0033 (11.5 ± 0.5)	0.1	0.11
0.0002 (12.7)	0.26 (9.6 ± 0.4)	0.2	0.17	0.1 (10.0)	0.00039 (12.4 ± 0.5)	0.01	0.019
0.002 (11.7)	0.39 (9.4 ± 0.2)	0.3	0.31	1 (9.0)	0.000058 (13.2 ± 0.4)	0.002	0.066

The 50% effective concentration (EC_{50}) is the range determined from at least three independent experiments. pEC_{50} , and pConc are log-transformed EC_{50}, and Conc., respectively. All data represent the mean ± standard deviation, n = 4–6. †Combination index (C.I.) is calculated from the following equation: C.I. = $D_A/D_{50,A}+D_B/D_{50,B}$. Where D_A and D_B are the concentrations of compound A and B that achieve a 50% effective concentration under drug combination, and $D_{50,A}$ and $D_{50,B}$ denote the EC_{50} values under monotherapy. C.I. < 1.0 denotes synergism, C.I. = 1.0 denotes an additive effect and C.I. > 1.0 denotes antagonism.

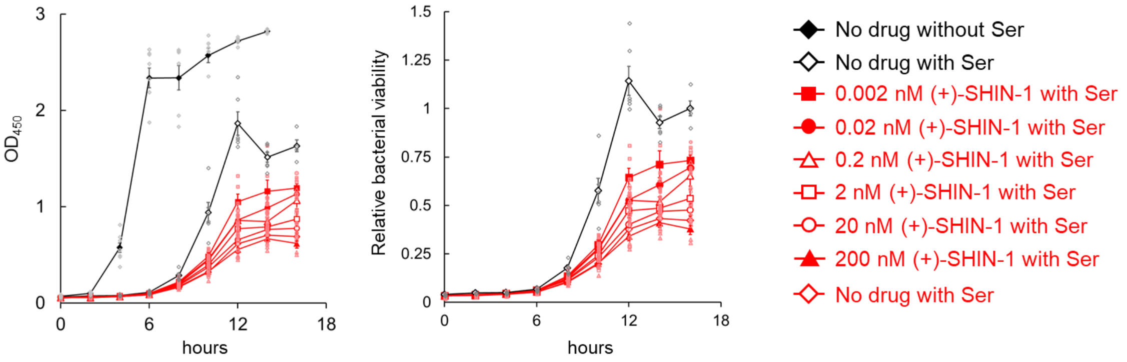

Supplementary Fig. S1 Effect of glycine on *E. faecium* growth. a) OD_{450} values were shown. A hundred molar of Ser suppressed the growth of *E. faecium*. b) Relative bacterial viabilities were shown. EC_{50} value at 16 hours later was between 2 and 20 nM. These experiments were performed six times. Error bars mean standard error. Gray and light red-colored symbols are the values for calculating the average values and standard error of each point (n=4 at 16 hours and n=6 at other data points).

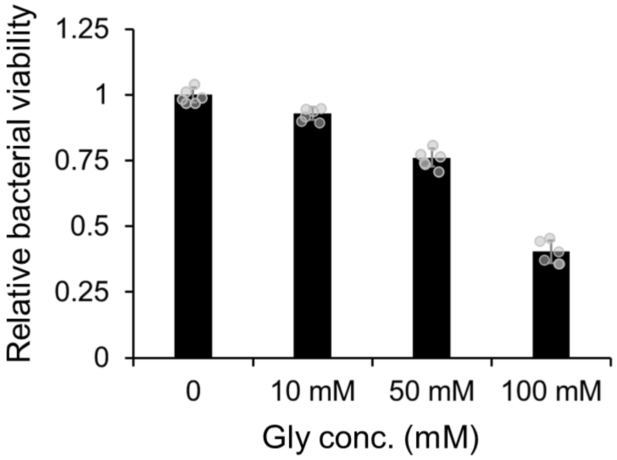

Supplementary Fig. S2 Cell cytotoxic effect of glycine. In the presence of 50 and 100 mM glycine, relative bacterial viability decreased to ~0.75 and 0.40, respectively. Error bars mean standard error. Gray-colored circles are the values for calculating the average values and standard error (n=6).

a

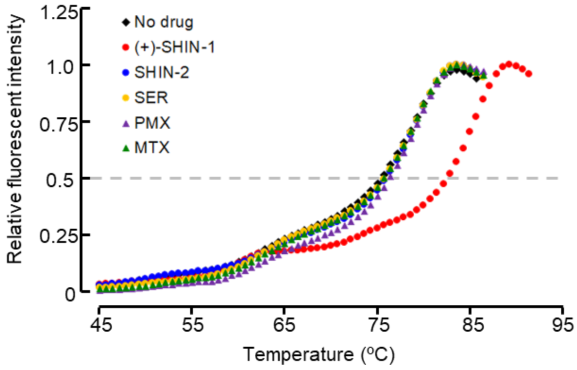

b

Compounds	T_m ± SD (ºC)	ΔT_m (ºC)
No drug	75.4 ± 0.5	0.0
(+)-SHIN-1	82.4 ± 0.2	7.0
SHMT-IN-2	75.9 ± 0.3	0.5
SER	75.8 ± 0.4	0.4
PMX	76.3 ± 0.2	0.9
MTX	75.6 ± 0.4	0.2

Supplementary Fig. S3 Thermal stability of *efm*SHMT with/without each compound as determined using DSF. a Thermal denaturation, which was detected using SYPRO Orange in *efm*SHMT without drug (black diamond) and with (+)-SHIN-1 (red circle), SHMT-IN-2 (blue circle), SER (yellow circle), PMX (purple triangle) and MTX (green triangle). (b) T_m values (the temperature at which the relative fluorescent intensity is 0.5) for each *efm*SHMT-inhibitor complex. All data represent the mean ± standard deviation (n=3).

a

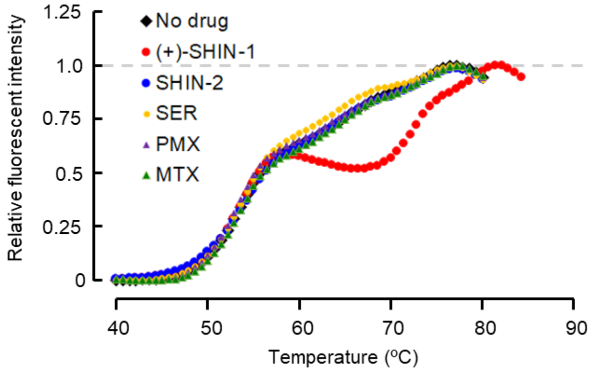

b

Compounds	T_{max} ± SD (ºC)	ΔT_{max} (ºC)
No drug	76.9 ± 0.4	0.0
(+)-SHIN-1	81.5 ± 0.0	4.6
SHMT-IN-2	77.2 ± 0.0	0.3
SER	76.8 ± 0.4	-
PMX	77.2 ± 0.5	0.3
MTX	77.2 ± 0.0	0.3

Supplementary Fig. S4 Thermal stability of *ec*SHMT in the absence or presence of each compound, as determined using DSF. **a** Thermal denaturation was detected using SYPRO Orange. *ec*SHMT in the absence of a drug (black diamond) and in the presence of (+)-SHIN-1 (red circle), SHMT-IN-2 (blue circle), SER (yellow circle), PMX (purple triangle) and MTX (green triangle). **b** T_{max} values (the temperature at which the relative fluorescent intensity value was 1.0) for each *efm*SHMT complex. All data represent the mean ± standard deviation (n=3).

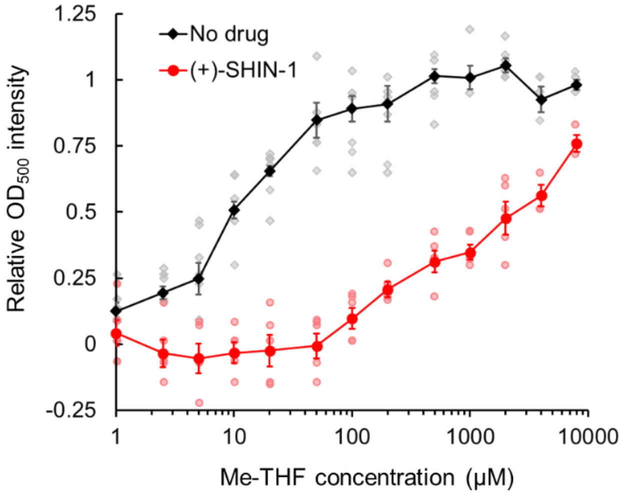

Supplementary Fig. S5 OD_{500} values with each concentration of (+)-SHMT and 2.5 μM efmSHMT. Gray- and light red-colored symbols show the values for calculating the average values and standard error (SE) of each point (n=4 and 3 at 4000 and 8000 μM of Me-THF, respectively, and n=6 at other data points).

a

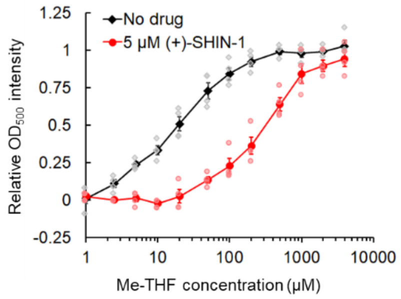

b

Compounds	SHMT	Me-THF	Compounds	
	Conc. (µM) (pConc.)	EC_{50} ± SE (µM) (pEC_{50} ± SE)	Conc. (µM) (pConc.)	K_i† ± SE (µM) pK_i ± SE
No drug	5 (5.3)	21 (4.7 ± 0.1)	-	-
(+)-SHIN-1	5 (5.3)	310 (3.5 ± 0.1)	5 (5.3)	0.31 (6.5 ± 0.1)

Supplementary Fig. S6 Competitive binding assay results. a Relative OD_{500} at each concentration of Me-THF and 5 µM *efm*SHMT in the presence and absence of (+)-SHIN-1. All experiments were carried out under conditions with 10 mM Gly but without Ser. The binding affinity of (+)-SHIN-1 to *efm*SHMT was weaker than that observed in the presence of Ser. These experiments were performed four times. pEC_{50} , pConc and pK_i are log-transformed EC_{50}, Conc. and K_i, respectively. Error bars mean standard error. Gray and light red-colored symbols are the values for calculating the average values and standard error of each point (n=4).

Supplemental Reference

1. Dutka-Malen, S., Evers, S. & Courvalin, P. Detection of glycopeptide resistance genotypes and identification to the species level of clinically relevant enterococci by PCR. Journal of clinical microbiology 33, 24-27 (1995).
