# Supplementary material for: Serine hydroxymethyltransferase as a potential target of antibacterial agents acting synergistically with one-carbon metabolism-related inhibitors
Source: Commun Biol. 2022 Jun 23;5:619. doi: 10.1038/s42003-022-03555-x (PMC9223267; doi:10.1038/s42003-022-03555-x)
Supplement: Supplementary file 3 — Description of Additional Supplementary Files [file 42003_2022_3555_MOESM3_ESM.pdf]

Description of Additional Supplementary Files

File name: Supplementary Data 1
Description: the source data behind the graph in Fig. 2.

File name: Supplementary Data 2
Description: the source data behind the graph in Fig. 3b.

File name: Supplementary Data 3
Description: the source data behind the graph in Fig. 4a.

File name: Supplementary Data 4
Description: the source data behind the graph in Fig. 7.
