# Supplementary material for: Serine hydroxymethyltransferase as a potential target of antibacterial agents acting synergistically with one-carbon metabolism-related inhibitors
Source: Commun Biol. 2022 Jun 23;5:619. doi: 10.1038/s42003-022-03555-x (PMC9223267; doi:10.1038/s42003-022-03555-x)
Supplement: Supplementary file 8 — Reporting Summary [file 42003_2022_3555_MOESM8_ESM.pdf]

nature portfolio

Corresponding author(s): Hironori Hayashi

Last updated by author(s): May 1, 2022

Reporting Summary

Nature Portfolio wishes to improve the reproducibility of the work that we publish. This form provides structure for consistency and transparency in reporting. For further information on Nature Portfolio policies, see our Editorial Policies and the Editorial Policy Checklist.

Statistics

For all statistical analyses, confirm that the following items are present in the figure legend, table legend, main text, or Methods section.

n/a	Confirmed	
☐	☒	The exact sample size (*n*) for each experimental group/condition, given as a discrete number and unit of measurement
☒	☐	A statement on whether measurements were taken from distinct samples or whether the same sample was measured repeatedly
☒	☐	The statistical test(s) used AND whether they are one- or two-sided<br>*Only common tests should be described solely by name; describe more complex techniques in the Methods section.*
☒	☐	A description of all covariates tested
☒	☐	A description of any assumptions or corrections, such as tests of normality and adjustment for multiple comparisons
☐	☒	A full description of the statistical parameters including central tendency (e.g. means) or other basic estimates (e.g. regression coefficient) AND variation (e.g. standard deviation) or associated estimates of uncertainty (e.g. confidence intervals)
☒	☐	For null hypothesis testing, the test statistic (e.g. *F*, *t*, *r*) with confidence intervals, effect sizes, degrees of freedom and *P* value noted<br>*Give P values as exact values whenever suitable.*
☒	☐	For Bayesian analysis, information on the choice of priors and Markov chain Monte Carlo settings
☒	☐	For hierarchical and complex designs, identification of the appropriate level for tests and full reporting of outcomes
☒	☐	Estimates of effect sizes (e.g. Cohen's *d*, Pearson's *r*), indicating how they were calculated

Our web collection on statistics for biologists contains articles on many of the points above.

Software and code

Policy information about availability of computer code

Data collection	SPring-8 BL32XU and BL41XU
Data analysis	Phenix (version 1.13_2998), Phaser (version 2.8.1/2.8.3), CCP4i (version 6.5), COOT (version 0.8.9.3-pre), PyMOL (version 1.4.1), Excel (version 2102)

For manuscripts utilizing custom algorithms or software that are central to the research but not yet described in published literature, software must be made available to editors and reviewers. We strongly encourage code deposition in a community repository (e.g. GitHub). See the Nature Portfolio guidelines for submitting code & software for further information.

Data

Policy information about availability of data

All manuscripts must include a data availability statement. This statement should provide the following information, where applicable:

- Accession codes, unique identifiers, or web links for publicly available datasets
- A description of any restrictions on data availability
- For clinical datasets or third party data, please ensure that the statement adheres to our policy

Crystal structure data that support the findings of this study have been deposited in Protein Data Bank with the PDB ID: 7V3D, 7X5N and 7X5O. All relevant data supporting the findings in this study are provided from the Source Data file or the corresponding author upon reasonable request.

Field-specific reporting

Please select the one below that is the best fit for your research. If you are not sure, read the appropriate sections before making your selection.

☒ Life sciences ☐ Behavioural & social sciences ☐ Ecological, evolutionary & environmental sciences

For a reference copy of the document with all sections, see nature.com/documents/nr-reporting-summary-flat.pdf

Life sciences study design

All studies must disclose on these points even when the disclosure is negative.

Sample size	Statistical methods were not used to determine sample sizes.<br>However, for quantification purposes, a sample size of n > 3 unless otherwise started (reproduced and confirmed by conducting the experiments on three or more independent occasions) was used. Sample size were determined based on prior experiences in the field.
Data exclusions	No data were excluded.
Replication	To ensure reproducibility of experimental findings, each assay was performed at least three times to confirm the results.
Randomization	Randomized for experiments was not relevant because all agents, bacteria and cells used for analysis wee from the same initial stocks.
Blinding	Blinding for experiments was not relevant because all data collection and analysis were quantitative and not qualitative in nature.

Reporting for specific materials, systems and methods

We require information from authors about some types of materials, experimental systems and methods used in many studies. Here, indicate whether each material, system or method listed is relevant to your study. If you are not sure if a list item applies to your research, read the appropriate section before selecting a response.

Materials & experimental systems

n/a	Involved in the study	
☒	☐	Antibodies
☐	☒	Eukaryotic cell lines
☒	☐	Palaeontology and archaeology
☒	☐	Animals and other organisms
☒	☐	Human research participants
☒	☐	Clinical data
☒	☐	Dual use research of concern

Methods

n/a	Involved in the study	
☒	☐	ChIP-seq
☒	☐	Flow cytometry
☒	☐	MRI-based neuroimaging

Eukaryotic cell lines

Policy information about cell lines

Cell line source(s)	Hep2 was kind gifts from Fukushima Medical University. Calu-3 were purchased from the American Type Culture Collection (ATCC, VA, USA). Caski was purchased from the Cell Resource Center for Biomedical Research (Cell Bank of Tohoku University).
Authentication	Authentication was performed by morphology check under microscopes.
Mycoplasma contamination	All cell lines used in the present study were tested negative for mycoplasma contamination.
Commonly misidentified lines<br>(See ICLAC register)	No commonly misidentified cell lines were used.
